# A Web-Based Tool for Quantification of Potential Gains in Life Expectancy by Preventing Cause-Specific Mortality

**DOI:** 10.3389/fpubh.2021.663825

**Published:** 2021-07-01

**Authors:** Aruna Chandran, Churong Xu, Jonathan Gross, Kathryn M. Leifheit, Darcy Phelan-Emrick, Stephane Helleringer, Keri N. Althoff

**Affiliations:** ^1^Department of Epidemiology, Johns Hopkins Bloomberg School of Public Health, Baltimore, MD, United States; ^2^Baltimore City Health Department, Baltimore, MD, United States; ^3^Department of Health Policy and Management, Fielding School of Public Health, Los Angeles, CA, United States; ^4^Department of Social Research and Public Policy, New York University, New York, NY, United States

**Keywords:** life expectancies, neighborhood, local health, mortality reduction, web-based tool

## Abstract

**Introduction:** Local health departments are currently limited in their ability to use life expectancy (LE) as a benchmark for improving community health. In collaboration with the Baltimore City Health Department, our aim was to develop a web-based tool to estimate the potential lives saved and gains in LE in specific neighborhoods following interventions targeting achievable reductions in preventable deaths.

**Methods:** The PROLONGER (Im**PRO**ved **LONGE**vity through **R**eductions in Cause-Specific Deaths) tool utilizes a novel Lives Saved Simulation model to estimate neighborhood-level potential change in LE after specified reduction in cause-specific mortality. This analysis uses 2012–2016 deaths in Baltimore City residents; a 20% reduction in heart disease mortality is shown as a case study.

**Results:** According to PROLONGER, if heart disease deaths could be reduced by 20% in a given neighborhood in Baltimore City, there could be up to a 2.3-year increase in neighborhood LE. The neighborhoods with highest expected LE increase are not the same as those with highest heart disease mortality burden or lowest overall life expectancies.

**Discussion:** PROLONGER is a practical resource for local health officials in prioritizing scarce resources to improve health outcomes. Focusing programs based on potential LE impact at the neighborhood level could lend new information for targeting of place-based public health interventions.

## Introduction

Local health departments play a fundamental role in delivering public health services and frequently employ neighborhood-based strategies to allocate interventions in order to maximize overall population health using limited resources (1). In US cities, population health profiles vary tremendously by neighborhood, shaped by the intersection of socio-political history, natural and built environment, and population features (2).

Life expectancy (LE) is a core measure of the overall health status of a population. LE at birth is defined as the average number of years a newborn is expected to live, assuming the current mortality rates are unchanged over the newborn's lifespan. Unfortunately, not all regions or subpopulations within the US experience the same LE (3, 4). Using Baltimore City, Maryland as an example, the average LE at birth in 2016 was 73.2 years, 5.7 years lower than the US's average of 78.9 and 5.9 years lower than Maryland's average of 79.1 years (4–6). Within Baltimore City's Community Statistical Areas (CSAs), geographic groupings of neighborhoods in the city, the 2016 LE ranged from 66.4 years in Downtown/Seton Hill, an inner-city neighborhood in West Baltimore, to 85.2 years in Cross-Country/Cheswolde, a neighborhood on the city's northern border approaching the wealthy suburban communities in Baltimore County. This represents a nearly 20-year LE gap between two neighborhoods in the same city, located just 6 miles apart (4).

Averting preventable deaths with interventions targeting modifiable or preventable risk factors is an actionable goal for local public health officials. Many local health departments are able to identify leading causes of preventable mortality at a neighborhood level. For example, in the 2017 Baltimore City Neighborhood Health Profiles, the Baltimore City Health Department (BCHD) highlighted CSA-level differences in rates of leading causes of death (7). The CSA of Downtown/Seton Hill had among the highest rates of breast cancer and stroke deaths, while the CSA of Cross-County/Cheswolde had high rates of mortality from prostate cancer and HIV. Comparing leading causes of mortality by neighborhood shifts the focus for community members and local health officials to think about how to target efforts to improve health in each community, rather than focusing exclusively on those communities with the poorest overall health indicators (8).

Local health departments are currently limited in their ability to use LE as a benchmark for improving community health. With a straightforward way to forecast LE gains to be expected from intervening on a cause of death, health departments could target evidence-based interventions to maximize potential lives saved and LE gained within neighborhoods. In collaboration with the BCHD, we developed a web-based tool named PROLONGER (Im**PRO**ved **LONGE**vity through **R**eductions in Cause-Specific Deaths) to estimate the potential lives saved and gains in life expectancy in specific neighborhoods following interventions targeting achievable reductions in preventable deaths. In this paper, we explain the methodology used in the development of this tool and illustrate its use with Baltimore City's cause-specific mortality data as a case-study.

## Methods

In this section, we describe the calculations and model specifications used in the web-based tool, as well as details about the setting (Baltimore City, Maryland) in which we demonstrate this tool. All calculations were done using R Version 3.5.2, and the tool was developed using the RShiny package. This project was reviewed by the Institutional Review Board of the Johns Hopkins Bloomberg School of Public Health and determined not to constitute Human Subjects Research.

### Mortality, Population, and Observed Life Expectancy

To use the PROLONGER tool, an end-user must upload mortality data, either at the individual-level or aggregated by geographic unit. These data are usually obtained from a city or state vital statistics department. Users choosing to upload individual-level (de-identified) data would need to include the following variables: year of death, age at death, ICD-10 cause of death, and geographic unit of residence at time of death. Users seeking information disaggregated by sex and/or race would need to include those categories as well. Understanding that many end-users may not have access, permission, or feel comfortable uploading individual-level mortality data, we demonstrate aggregated (by year and 5-year age categories until age 85+) mortality data is a feasible alternative.

Population estimates by age category are necessary for calculating mortality rates and life expectancy estimates. CSAs in Baltimore City are aligned with US Census Tracts. Therefore, the tool uses annual population estimates developed by the US Census Bureau from the annual American Community Surveys, which are available disaggregated by sex, race and age category at the Census Tract level (9). If a user is interested in obtaining LE estimates for neighborhoods in which boundaries do not align with those of the US Census Bureau, the user would need to upload population numbers by age category as well.

Observed at-birth LE is estimated by the tool using the Chiang methodology, shown to be optimal for the calculations of LE in small populations of 5,000 or less (10, 11).

### Proportion of Deaths Averted

For each cause of death, the tool provides lives saved and LE estimates resulting from averting 5, 10, 15, and 20% of a single cause of preventable deaths. These values were chosen as potentially achievable goals of mortality reductions using evidence-based screening and prevention strategies. The end-user can compare the potential impact of an intervention, as quantified by potential lives saved and LE gained, given different potential levels of mortality burden reduction from a chosen program.

When imposing a reduction in deaths, the optimal way to preserve the exact age distribution of disease-specific mortality would be to reduce deaths in each age category by the chosen percentage. However, this is not realistic from a programmatic perspective. For example, most cardiovascular disease prevention programs are not designed for implementation across all age groups equally, but rather are likely to target middle-aged individuals. Therefore, we compared imposing a reduction in deaths overall vs. within each age category using the *R*^2^ coefficient.

### Calculation of Lives Saved

In the tool, the **LI**ves **S**aved **S**imulati**O**n (LISSO) model is used to estimate the potential number of lives saved if success is achieved in reducing preventable deaths. Details of the development of the LISSO model have been published elsewhere (12). Briefly, we adapted the model employed by Case and Deaton to show potential changes in survival given aversion of cause-specific deaths while still allowing those survivors to age into older age categories and face the same subsequent mortality risk as their peers (13). Expected mortality rates after averting a proportion of cause-specific deaths were calculated by multiplying observed mortality rates by the proportion of lives saved for the index year. The individual lives saved were followed into subsequent years with removal of the proportion of those lives saved that would experience death from the same mortality rate as their peers in those years. Unique to the LISSO model is the inclusion of an agent-based 100-fold simulation step in which the specific individuals that survive are randomly selected. The median number of lives saved from the 100 simulations is used in the subsequent LE calculations.

### Calculation of Expected Life Expectancy

The tool then calculates expected at-birth LE following aversion of specified proportions of deaths (5, 10, 15 or 20%) using the same Chiang methodology (11).

The challenge of calculating LE at a neighborhood level is cells that have either 0 or a very small number of deaths attributable to a specific cause, which can artificially inflate the resulting LE. Therefore, all cells with <5 deaths were replaced with a predicted number of deaths calculated using a negative binomial regression model including the following CSA-level covariates for Baltimore City (derived from the US Census Bureau) (9): age at start of the age interval, total age-specific population, proportion of non-Hispanic Black/African American race, proportion of non-Hispanic white race, median household income, and proportion of educational attainment above high school. Less than 5 deaths per cell was chosen as the appropriate cut-off for replacement in line with the CDC's life expectancy calculation methods, in which smoothing techniques are applied to estimate similarly low observed numbers of deaths (14). In cases where the predicted number was greater than the total number of deaths due to the selected cause in that age category, the number was replaced with the total number of deaths.

### Individual vs. Aggregate-Level Death Data

We anticipate that, given laws protecting personal health information, local health departments or agencies may not want to upload de-identified individual-level vital statistics data to a web-based tool. The LISSO model was developed for individual-level data, as the model simulates a random selection of specific individuals to “survive” for each estimate of lives saved. In order to accommodate both individual-level as well as aggregate-level death data, we made the following modification in the PROLONGER tool.

With aggregate level data, we made the conservative assumption that the deaths in that age category occurred in a uniform distribution across the 5-year age range. For race and/or sex disaggregated LE calculations, the race and sex distributions are allocated to age groups in the same stratum-specific proportions as the deaths in that age group; within each age group, the deaths are once again distributed uniformly. To test the assumption that aggregate and individual-level data result in similar LE calculations, we calculated R-squared coefficients relating predicted deaths and LE produced from the aggregate models to estimates from the individual-level models.

### Tool Interface

The tool interface provides users with tabbed selections of whether they would like to estimate potential LE gains for various causes of mortality in a specific geographic location, or if they would like to select a specific cause of mortality to target and have the tool select the locations that would have the greatest LE benefit. Within each tab, the user selects whether they have individual-level or aggregate-level data, and then is able to choose the percent of cause-specific deaths they believe their intervention would prevent. In addition, the user is able to select whether they would like to see the LEs stratified by sex and/or race.

The tool provides results in tabular form, as well as bar graphs and maps, available for export by the end-user. Finally, there is a tab within the tool detailing the methodology and code used to create the tool.

### Case Study: Geographic Area

Because the boundaries of a “neighborhood” can differ by personal opinion, the Baltimore Data Collaborative and the Baltimore City Department of Planning systematically developed boundaries for 55 Community Statistical Areas (CSAs) with Baltimore City, Maryland for which sub-city level data could be calculated. Each CSA contains 1–8 demographically homogenous Census Tracts, with total populations of 5,000–20,000 residents. The CSA boundaries were initially developed in 2000, and last modified in 2010. These are the neighborhood units used by BCHD for sub-city level publications such as the Neighborhood Health Profiles, as well as by several other organizations publishing sub-city level information (4, 7).

### Case Study: Deaths

Information from all death certificates filed for Baltimore City residents is compiled by the Maryland Vital Statistics Administration. Vital statistics data for Baltimore City are released annually to the Baltimore City Health Department. Study co-investigators based at the Baltimore City Health Department ran the tool's code using individual- and aggregate-level vital statistics data on site. All Baltimore City deaths that occurred between 2012 and 2016 that are assigned a census tract of residence and have an ICD-10 code for cause of death were included in this analysis. The Maryland Vital Statistics Administration (VSA) codes all reported deaths by cause using the 10th revision of the International Classification of Disease (ICD-10) system (6).

### Case Study: Leading Preventable Causes of Death

In the Baltimore City 2017 Neighborhood Health Profiles, the leading underlying causes of death included heart disease (24.4% of all Baltimore City deaths), cancer (all kinds, 21.3%), lung cancer (5.9%), stroke (4.9%), drug- and/or alcohol-Induced (4.5%), chronic lower respiratory disease (3.5%), accident (3.5%), homicide (3.5%), diabetes (3.0%), septicemia (2.7%), colorectal cancer (2.0%), HIV/AIDS (1.8%), breast cancer (1.5%), prostate cancer (1.1%) (7).

## Results

### Model Specifications

#### Overall vs. Within-Age-Group Reduction in Deaths

When imposing a reduction in deaths, the optimal way to preserve the exact age distribution of disease-specific mortality would be to reduce deaths within each age category by the chosen percentage. However, this is not realistic from a programmatic perspective. For example, most cardiovascular disease prevention programs are not designed for implementation across all age groups equally, but rather are likely to target middle-aged individuals. Therefore, we quantified the difference between imposing a 20% reduction in heart disease deaths, comparing a reduction to the overall number vs. applying the 20% reduction within each age group. Both the total deaths as well as the calculated expected LE were extremely similar using both methods (*R*^2^ = 1).

#### Aggregate vs. Individual-Level Data

We tested whether the model would yield similar results if a health department were to upload aggregate death tables as opposed to individual-level data. In order to recreate the simulation for which lives were saved when aggregate death tables were used, the tool creates a pseudo-dataset under the conservative assumption that all deaths within an age group are evenly distributed across that 5-year age group. Using a 20% reduction in heart disease deaths as the test, the calculated expected LEs were compared with aggregate and individual data. The *R*^2^ between expected number of deaths using individual-level vs. aggregate-level data was 0.999. When comparing expected LEs, the *R*^2^ was 0.998. Thus, there was a high degree of correlation in both expected number of deaths and expected LEs whether calculated using aggregate or individual-level data.

## Case Study: Baltimore City Findings

For this case study, we examined applying the PROLONGER tool to Baltimore City, Maryland, using a 20% reduction in heart disease mortality. Interestingly, the neighborhoods where we see the greatest potential LE increases from reductions in heart disease mortality were not the same neighborhoods identified purely by burden, demonstrating the power of the PROLONGER tool to inform the allocation of resources to maximize population health gains.

**Figure 1:**
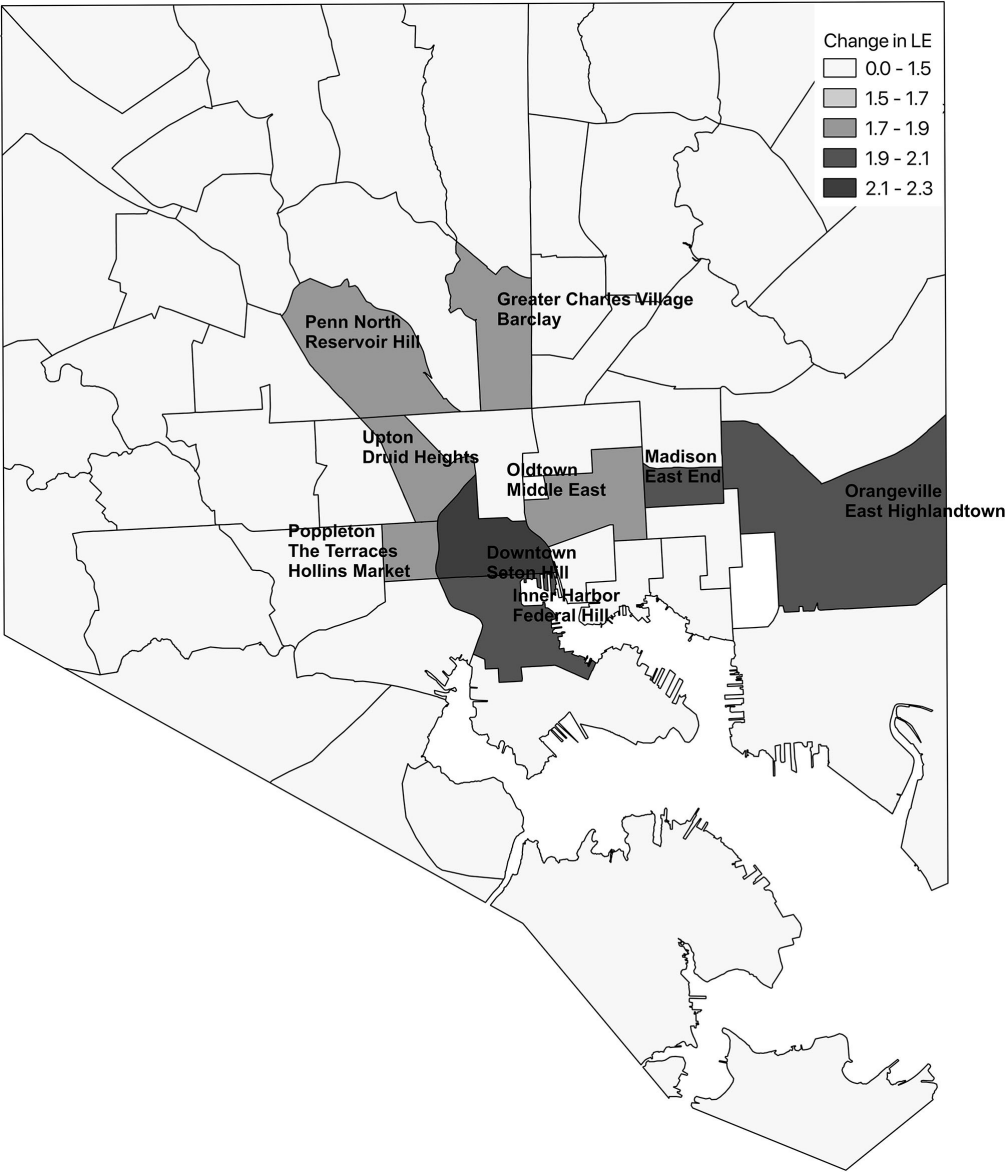
Ten Baltimore city community statistical areas (CSAs) with highest crude heart disease mortality **(A)** and ten CSAs with greatest potential increase in life expectancy following a 20% reduction in heart disease deaths **(B)**.

As with most other areas in the United States, heart disease is the leading cause of death in Baltimore City. Table 1 shows the ten CSAs with the highest crude heart disease mortality rates annualized over the 2012–2016 time period. The highest heart disease mortality rates of 4.3 per 1,000 occurred in Morrel Park/Violetville and Pimlico/Artlington/Hilltop (Figure 1A).

**Table 1:** Ten Baltimore city community statistical areas (CSAs) with the highest crude heart disease mortality rates (per 1,000 population)–2012–2016.

**CSA**	**Crude heart disease mortality rate (per 1,000 population)**
Morrell Park/Violetville	4.3
Pimlico/Arlington/Hilltop	4.3
Clifton-Berea	4.0
Greenmount East	3.8
Greater Rosemont	3.7
Greater Mondawmin	3.6
Oldtown/Middle East	3.5
Edmondson Village	3.4
Howard Park/West Arlington	3.4
Dorchester/Ashburton	3.3

Figure 1B shows the 10 CSAs with the largest expected increase in LE following a 20% reduction in heart disease in each CSA along with their observed LE. Highlandtown, Orangeville/East Highlandtown, Inner Harbor/Federal Hill, and Greater Charles Village/Barclay emerged as the best targets for overall interventions aimed to reduce heart disease based on the greatest potential increase in LE. This is in stark contrast to the CSAs that would be targeted for intervention based on heart disease mortality rates alone (Figure 1A). Morrel Park/Violetville and Pimlico/Artlington/Hilltop, which have the highest heart disease mortality rates, are not even in the top 10 of the expected change in LE following implementation of this hypothetical intervention. Following a 20% reduction in heart disease in each CSA, the median change in LE was 1.35 (IQR: 1.15, 1.58) years.

**Figure 2:**
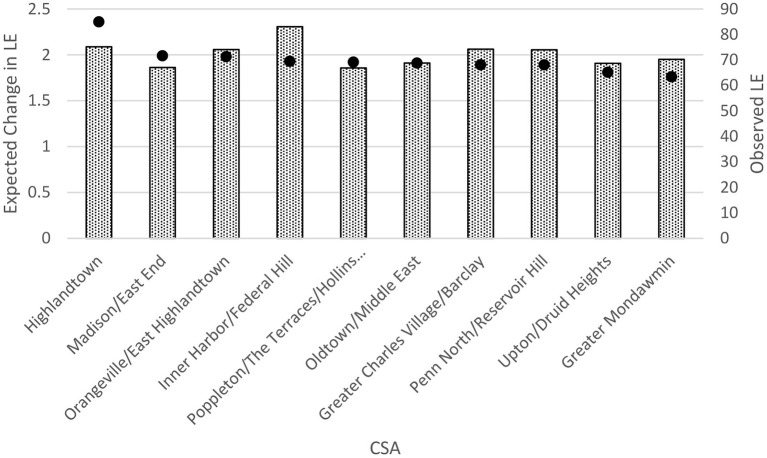
Ten Baltimore city community statistical areas (CSAs) with greatest potential increase in life expectancy (LE) following a 20% reduction in heart disease deaths compared with observed LE in those CSAs. The bars represent the observed LEs, and the dots represent the expected change between expected LE and observed LE.

Notably, it was not always the CSA with the lowest observed LE that would expect the greatest change in LE with a hypothesized 20% reduction in heart disease mortality. Figure 2 shows that a nearly 2.5-year increase in LE could be expected in Highlandtown even though Highlandown's observed LE is higher than that of Madison/East End, where a smaller 2.0-year increase could be expected. It is true that the CSAs in which higher proportions of deaths due to a cause of interest occur in younger populations would expect greater LE benefits by preventing deaths from that cause. However, we note that those CSAs with an overall younger age distribution did not always emerge as the neighborhoods with the largest expected increases in LE from a 20% reduction in heart disease deaths. In fact, nearly half (49%) of Baltimore City's 55 CSAs had younger population distributions (i.e., lower proportions of the total population aged 65+ years) than Highlandtown and Inner Harbor/Federal Hill.

Similar results were noted when disaggregating the population by sex or race (data not shown).

## Discussion

Working collaboratively with the Baltimore City Health Department, we developed a web-based PROLONGER tool that calculates neighborhood-level LE given mortality and population data that is uploaded by the end-user. While there are other tools that will calculate small-area life expectancy across the US [i.e., USALEEP, the US Small-area Life Expectancy Estimates Project (15)], to our knowledge this is the only tool that will then calculate potential gains in life expectancy from a measured number of lives saved. This tool allows local health decision/local government makers to quantify potential LE gains from the targeted implementation of interventions to reduce cause-specific mortality by achievable amounts.

The most notable finding from the PRLONGER tool, as demonstrated by the example of heart disease mortality reduction in Baltimore City, is that identified neighborhoods for targeted effort to reduce disease-specific risk factors might change if neighborhoods were selected based on potential LE gain as opposed to mortality burden. In terms of selecting target neighborhoods for particular disease screening or prevention efforts, different neighborhoods may emerge as having the greatest potential for LE change based on the disease of focus. This would allow local health officials to modify their work and messaging away from “blaming” the neighborhoods with highest overall mortality to instead understanding that all neighborhoods have opportunities for optimization of health and well-being for the city as a whole.

This project has several limitations. First, it is important to keep in mind that potential LE gains are heavily influenced by deaths that occur at younger ages; therefore, neighborhoods with younger age distributions, and more specifically deaths within the younger age groups, may more frequently be selected as high-impact targets for interventions. Users will need to keep in mind how the age distributions of their neighborhoods differ and interpret results accordingly. Second, the tool only considers single-causes of mortality, so neighborhoods where many younger residents are dying from a variety of causes will not emerge as areas with the highest expected LE benefits. From a health equity perspective, this is meaningful, as these are often the most under-resourced neighborhoods, home to low-income households and people of color. Future iterations of such tools should be adapted to incorporate multiple causes of death, as well as other structural factors that may affect the predicted success rates of planned interventions. Third, we are limited by the quality of residence and cause of death coding that is available through the Vital Statistics data. Ongoing efforts should be made at every level to improve this work across the US. Finally, calculation of LE in small areas remains a challenge. In this study we aimed to align our estimates with those published by the National Center for Health Statistics. We continue to support all efforts to improve the calculation of LE in small areas.

## Public Health Implications

The PROLONGER tool can empower local health officials to quantify the potential health impacts of the focused implementation of screening and prevention efforts within their cities. Local health departments and local government could use the tool to set specific goals and justify allocation of resources, and to communicate the rationale behind their decisions to senior leadership and key stakeholders. Using such information in local decision-making may help to reduce mortality and LE inequities across the US.

## Data Availability Statement

The data analyzed in this study is subject to the following licenses/restrictions: data can be obtained following review and permission from the Baltimore City Health Department. Requests to access these datasets should be directed to Paul Overly; paul.overly@baltimorecity.gov.

## Author Contributions

AC conceptualized this study and oversaw all aspects of the project. CX created the tool and all code for the analysis. JG ran the code on the raw data. DP-E, SH, and KA provided valuable technical assistance throughout the process. All authors participated in the preparation of the manuscript.

## Conflict of Interest

The authors declare that the research was conducted in the absence of any commercial or financial relationships that could be construed as a potential conflict of interest.
